# Antitumor Activity of Ferulic Acid Against Ehrlich Solid Carcinoma in Rats via Affecting Hypoxia, Oxidative Stress and Cell Proliferation

**DOI:** 10.7759/cureus.41985

**Published:** 2023-07-17

**Authors:** Mohammad A Alghamdi, Talal A Khalifah, Hisham S Alhawati, Mazen Ruzayq, Abdullah Alrakaf, Ahmed Khodier, Mohammed M Al-Gayyar

**Affiliations:** 1 Pharmacology, University of Tabuk, Tabuk, SAU; 2 Clinical Pharmacology, Delta University for Science and Technology, Gamasa, EGY; 3 Pharmaceutical Chemistry, University of Tabuk Faculty of Pharmacy, Tabuk, SAU; 4 Biochemistry, Mansoura University Faculty of Pharmacy, Mansoura, EGY

**Keywords:** signal transducer and activator of transcription 3 (stat3), nuclear factor erythroid 2–related factor 2 (nrf2), mammalian target of rapamycin (mtor), hypoxia-inducible factor (hif)-1α, heme oxygenase-1 (ho-1), ehrlich solid carcinoma (esc), cyclin d1, cellular myc (cmyc)

## Abstract

Background

Ferulic acid is a natural compound commonly found in fruits and vegetables like tomatoes, sweet corn, rice bran, and dong quai. It has various beneficial effects on the body, such as anti-inflammatory, anti-apoptotic, hepatoprotective, cardioprotective, and neuroprotective properties.

Aims

We conducted a study to investigate the antitumor activity of ferulic acid against Ehrlich solid carcinoma (ESC), specifically by affecting hypoxia-inducible factor (HIF)-1α and its subsequent effects on other factors like nuclear factor erythroid 2-related factor 2 (Nrf2), heme oxygenase-1 (HO-1), cellular Myc (cMyc), cyclin D1, mammalian target of rapamycin (mTOR), and signal transducer and activator of transcription 3 (STAT3).

Materials and methods

The study involved implanting rats with ESC cells and administering 50 mg/kg of ferulic acid orally daily for eight days. Sections of the muscles with ESC were stained with toluidine blue or immunostained with anti-HIF-1α antibodies. The tumor samples were used to evaluate the expression of HIF-1α, Nrf2, HO-1, cMyc, cyclin D1, mTOR, and STAT3.

Results

Ferulic acid increased mean survival time, reduced tumor volume and weight, and improved the appearance of the tumor tissue. Furthermore, ferulic acid significantly elevated the expression of Nrf2 and HO-1, while reducing the expression of HIF-1α, Nrf2, HO-1, cMyc, cyclin D1, mTOR, and STAT3.

Conclusions

Ferulic acid can reduce tumor size and weight while improving the structure of muscle cells, suggesting it may have antineoplastic activity against ESC. Further investigation revealed that ferulic acid downregulates HIF-1α, increasing the expression of antioxidant proteins Nrf2 and HO-1. Additionally, ferulic acid decreases the expression of proliferation markers cMyc and cyclin D1 and downregulates cellular regulators mTOR and STAT3.

## Introduction

The Ehrlich cell is a mammary adenocarcinoma cultured in vivo, making it an ideal subject for biochemical studies requiring ample tissue samples. The cells are sustained through intraperitoneal passages in an ascitic form and can also manifest as solid tumors when injected subcutaneously [1]. Notably, this tumor's undifferentiated solid form makes it particularly valuable for tumor research. As a result, it is frequently utilized in chemotherapy studies and tumor model development.

There is a growing interest in developing novel drugs derived from natural plant-based compounds to serve as an alternative to traditional cancer treatments. These substances are often more effective and less toxic than conventional chemotherapy methods. Dong quai, a popular natural product in East Asia, is a member of the Apiaceae family and is widely used in traditional Chinese medicine. Its root can be ingested orally or applied topically as a cream to treat many conditions [2].

Ferulic acid is a type of natural compound that can be found in various fruits and vegetables, including sweet corn, tomatoes, dong quai, and rice bran. Research has shown that this phytochemical possesses several beneficial effects, such as antioxidants, antihyperglycemic, and hypolipidemic properties [3]. Additionally, studies indicate that ferulic acid can be an effective and safe adjunctive therapy for managing epilepsy-related depression when combined with levetiracetam [4]. Furthermore, it has been found to help prevent toxicity caused by anticancer drugs [5].

Some studies have reported that ferulic acid possesses the ability to treat cancer by impacting various mechanisms such as apoptosis [6], cell cycle arrest and autophagy [7], and inhibition of metastasis [8]. However, prior research has not yet explored the antitumor activity of ferulic acid concerning the oxygen-dependent transcriptional activator, hypoxia-inducible factor (HIF)-1α, and its subsequent effects on nuclear factor erythroid 2-related factor 2 (Nrf2) and heme oxygenase-1 (HO-1), which are antioxidant proteins. Additionally, we aim to examine its effect on cellular Myc (cMyc) and cyclin D1, which are proliferation markers. Finally, we will investigate the impact of ferulic acid on cellular regulators like the mammalian target of rapamycin (mTOR) and signal transducer and activator of transcription 3 (STAT3).

## Materials and methods

Animals and treatment outlines

Thirty-six Sprague Dawley rats weighing 180-200 g were used. They were kept in standard temperature conditions with a regular 12h light/12h dark cycle. The Research Ethics Committee of Horus University’s Faculty of Pharmacy approved the working protocol under number P2023-003. Rats were divided into three groups with 12 rats each.

The Control Group

Rats were kept without any treatment for the whole period of the experiment. 

ESC Group

Rats were subjected to intramuscular injection of 0.15 ml Ehrlich cells (2×10^6^) in the thigh of the left hind leg [9,10].

ESC Treated With Ferulic Acid

After induction of ESC in rats and once a solid tumor appeared on day 14, the time taken for a palpable mass of tumor to appear depends on the amount and viability of the injected cells, rats were given 50 mg/kg ferulic acid by oral gavage and indicated as day 0. Rats were treated with ferulic acid daily for three weeks. 

Sample collection 

The whole tumor area was separated from the thigh of the left hind leg, measured, and weighed. Part of the muscle tissue was fixed in 10% buffered formalin and used for morphologic and immunohistochemistry investigation. Another part was homogenized in a 10-fold ice-cold sodium-potassium phosphate buffer (0.01 M, pH 7.4) containing 1.15% KCl. The supernatant was stored at -80°C.

Morphologic analysis and immunohistochemistry

Muscle tissues were cut into 5-micrometer sections and stained with toluidine blue. Another 5-micrometer thick paraffin sections were immune stained with monoclonal anti-HIF-1α (Sigma-Aldrich, St. Louis, MO, USA) at 4°C as described previously by our group [11-13].

Enzyme-linked immunosorbent (ELISA) assay 

Commercially available ELISA kits were used for assessment of HIF-1α, Nrf2, HO-1, cMyc, cyclin D1, mTOR and STAT3 (MyBioSource, Inc., San Diego, CA, USA) according to manufacturer’s instructions.

Quantitative real-time polymerase chain reaction (RT-PCR)

The gene expression of HIF-1α, Nrf2, HO-1, cMyc, cyclin D1, mTOR, and STAT3 mRNA levels in rat muscle tissue lysate performed as described previously by our group [14-17]. GAPDH was used as a housekeeping gene and internal reference control. The gene-specific PCR primers used are summarized in Table 1.

**Table 1:** The primer set used for detection of gene expression in rats.

Name		Sequence	Reference sequence
GAPDH	F: 5`-CCATCAACGACCCCTTCATT-3` R: 5`-CACGACATACTCAGCACCAGC-3`		NM_017008.4
Nrf2	F: 5′-CTCTCTGGAGACGGCCATGACT-3′ R: 5′-CTGGGCTGGGGACAGTGGTAGT-3′		NM_031789
HO-1	F: 5′-CACCAGCCACACAGCACTAC-3′ R: 5′-CACCCACCCCTCAAAAGACA-3′		NM_012580
cMyc	F: 5′-CCCTCAGTGGTCTTCCCCTA-3′ R: 5′-AAGCTACGCTTCAGCTCGTT-3′		NM_012603.2
Cyclin D1	F: 5′-TCGACGGCCATTACCAATCG-3′ R: 5′-CGCAGACCTCTAGCATCCAG-3′		X75207.1
mTOR	F: 5′-CTGCACTTGTTGTTGCCTCC-3′ R: 5′-ATCTCCCTGGCTGCTCCTTA-3′		NM_019906.2
STAT3	F: 5′-CTGAGGTACAATCCCGCTCG-3′ R: 5′-TCGGTCAGTGTCTTCTGCAC-3′		NM_012747.2

Statistical analysis

To present quantitative variables, we used the mean ± standard error. We evaluated the normality of the sample distribution using the Kolmogorov-Smirnov (K-S) test. For comparing groups, we used a one-way analysis of variance (ANOVA), followed by a post hoc Bonferroni correction test. All statistical analyses were conducted using SPSS version 20 (IBM Corp., Armonk, NY, USA). We defined statistical significance as P < 0.05.

## Results

Antitumor activity of ferulic acid in ESC

**Figure 1:**
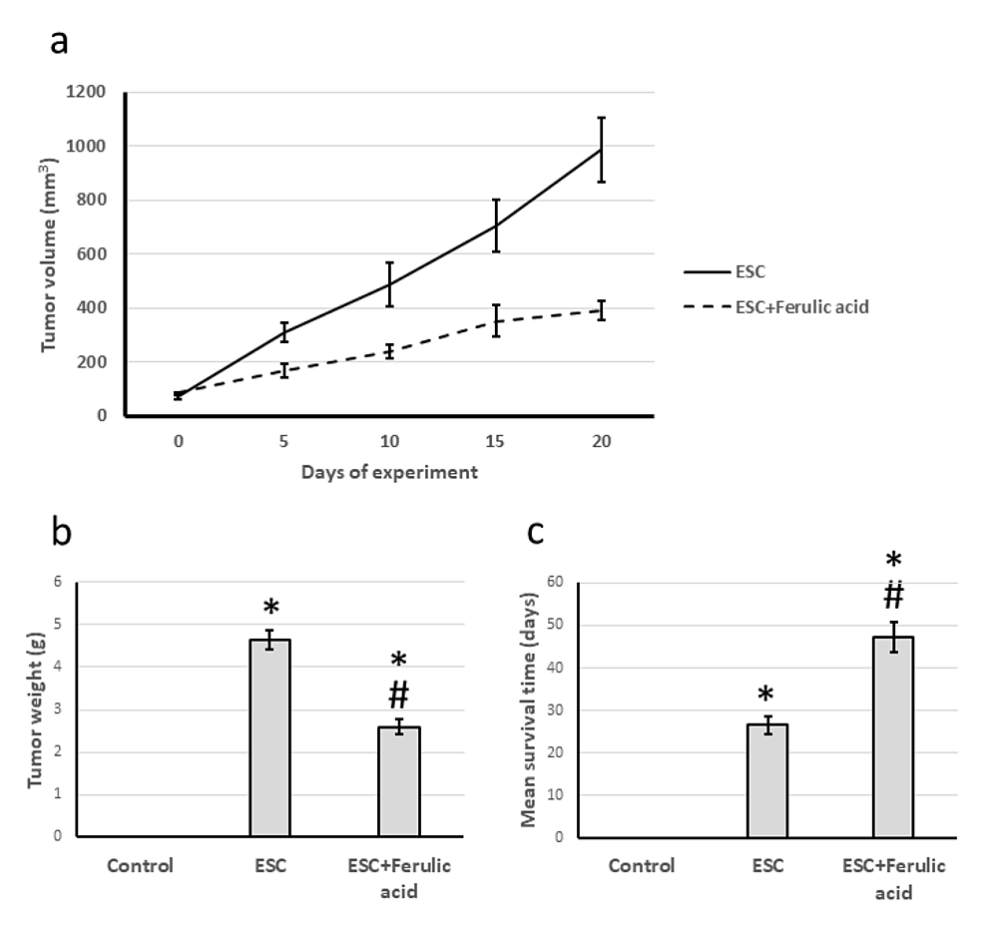
Effect of ESC and 50 mg/kg ferulic acid on tumor volume over the experiment period (a), tumor weight on the last day of the experiment (b), and rats’ mean survival time (c). * Represented a significant difference compared to the control group at a significance level of p<0.05. Similarly, # Represented a significant difference compared to the ESC group at a significance level of p<0.05. ESC, Ehrlich solid carcinoma.

During the experiment, there was a noticeable rise in tumor volume over time. Moreover, when compared to the control group, the tumor's weight showed a significant increase on the day of sacrifice. However, administering ferulic acid to ESC rats resulted in a significant reduction in both tumor volume and weight compared to the ESC group. Lastly, treating ESC with ferulic acid caused a noteworthy increase in mean survival time, extending it from 27 days to as much as 49 days (Figure 1).

Effect of ferulic acid on ESC-induced alteration in cells morphology

**Figure 2:**
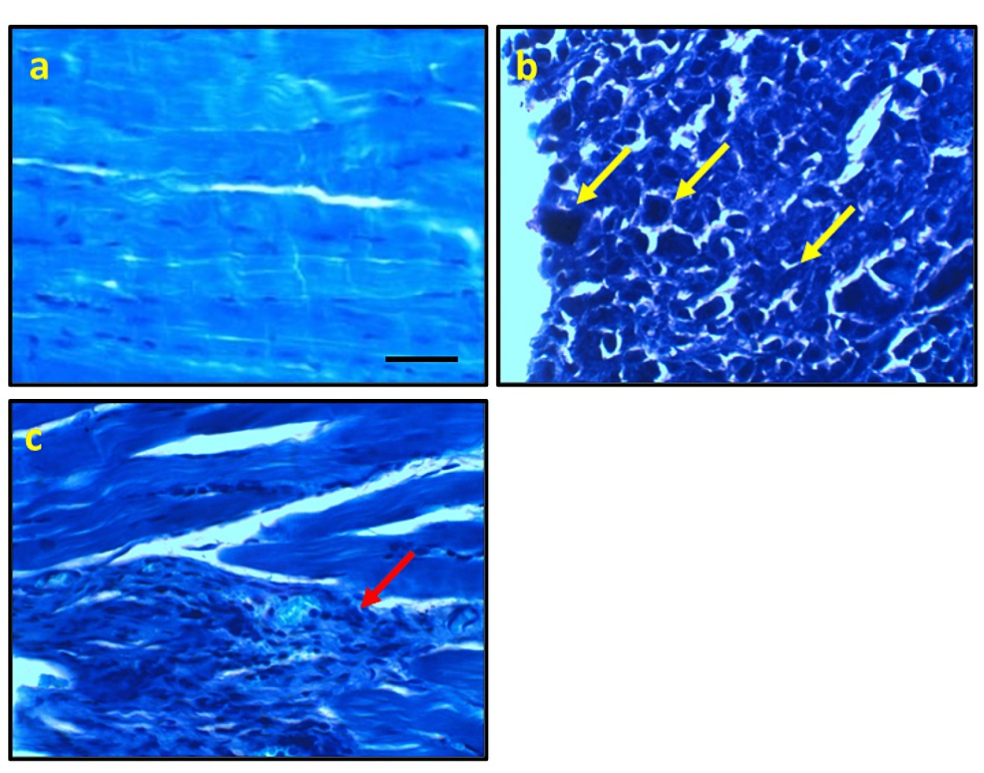
Representative photomicrograph stained with toluidine blue from the control group showing the normal histological appearance of muscle fiber nucleus (a), ESC showing delineated the diffuse infiltrative positive staining malignant cells with high nuclear-cytoplasmic ratio, clumped, roped, and stripping chromatin (yellow arrows, b). Ferulic acid improves the cell structure with few focal aggregations of positive stained round cells around muscle fibers (red arrows, c). * significant difference against the control group at p<0.05. # significant difference against the ESC group at p<0.05. Scale bar 100 μm. ESC, Ehrlich solid carcinoma.

Upon examining micro-images of control rats stained with toluidine blue, muscle fiber nuclei appeared typical in appearance. However, micro-images of ESC rats revealed malignant cells with high nuclear-cytoplasmic ratio, clumped chromatin, and stripling chromatin, which were delineated, diffuse, infiltrative, and positively stained. On the other hand, rats treated with ferulic acid showed an improvement in cell structure, albeit with a few focal aggregations of positively stained round cells around muscle fibers (Figure 2).

Effect of ferulic acid on ESC-induced elevation in the expression of HIF-1α

**Figure 3:**
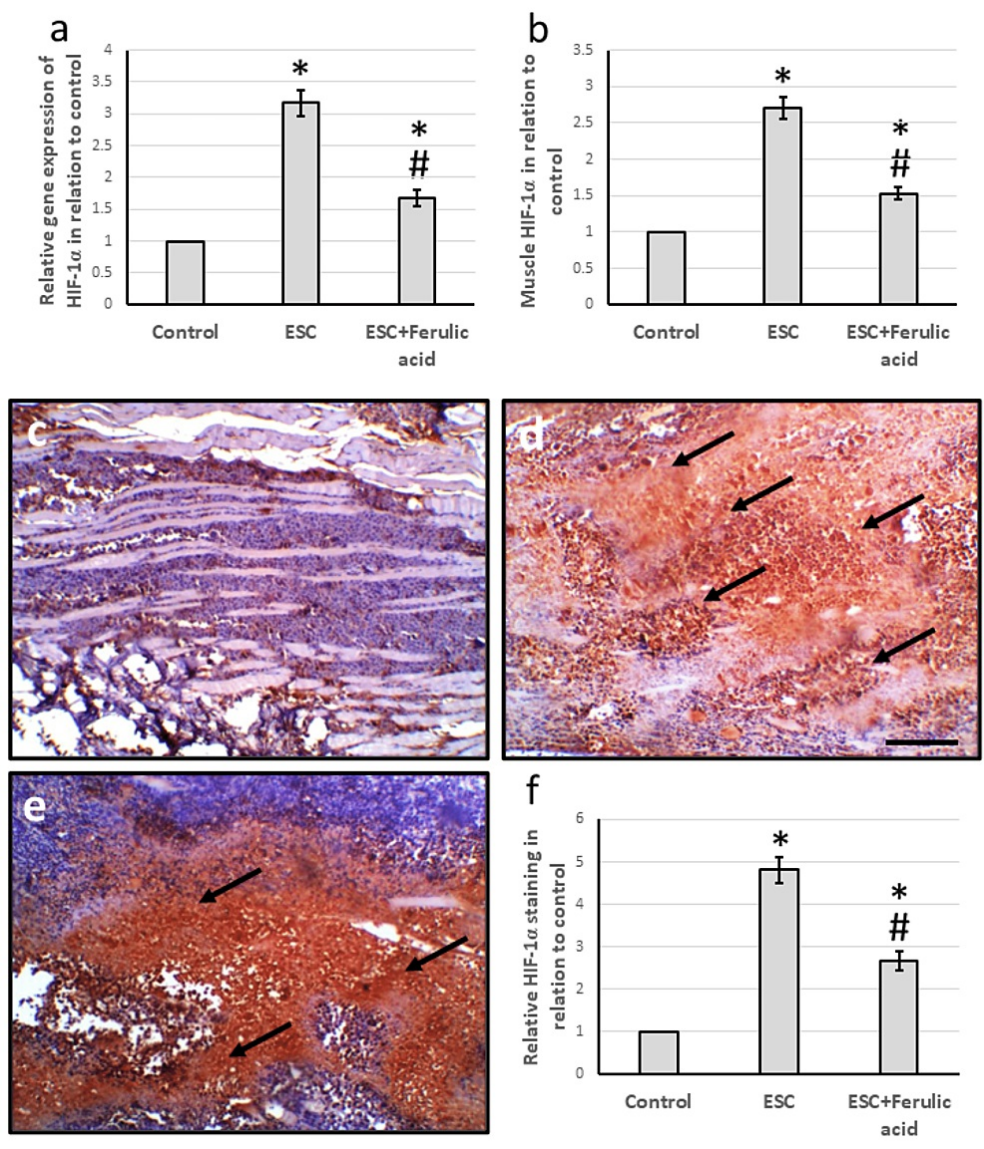
Effect of ESC and 50 mg/kg ferulic acid on gene expression of HIF-1α (a) and its protein level (b). Muscle sections stained with anti-HIF-1α antibodies in the control group (c), ESC (d), and ESC group treated with ferulic acid (e). Immunostaining score of positive staining (f). To calculate the immunohistochemistry score, we measured the total area stained with anti-Ki67 and divided it by the total tissue area in the field. We did this measurement in 10 different fields of each animal section. * significant difference against the control group at p<0.05. # significant difference against the ESC group at p<0.05. Scale bar 100 μm. ESC, Ehrlich solid carcinoma; HIF-1α, hypoxia-inducible factor.

The study found that ESC led to a significant increase in HIF-1α gene expression by 3.17 times and an increase in muscle levels by 2.71 times compared to the control group. Micro-images of muscle tissues stained with anti-HIF-1α also showed intense immune staining in the ESC group, which was confirmed by the relative area of staining with HIF-1α. However, these effects were reversed when ESC rats were treated with ferulic acid (Figure 3).

Effect of ferulic acid on ESC-induced reduction in the expression of Nrf2 and HO-1

**Figure 4:**
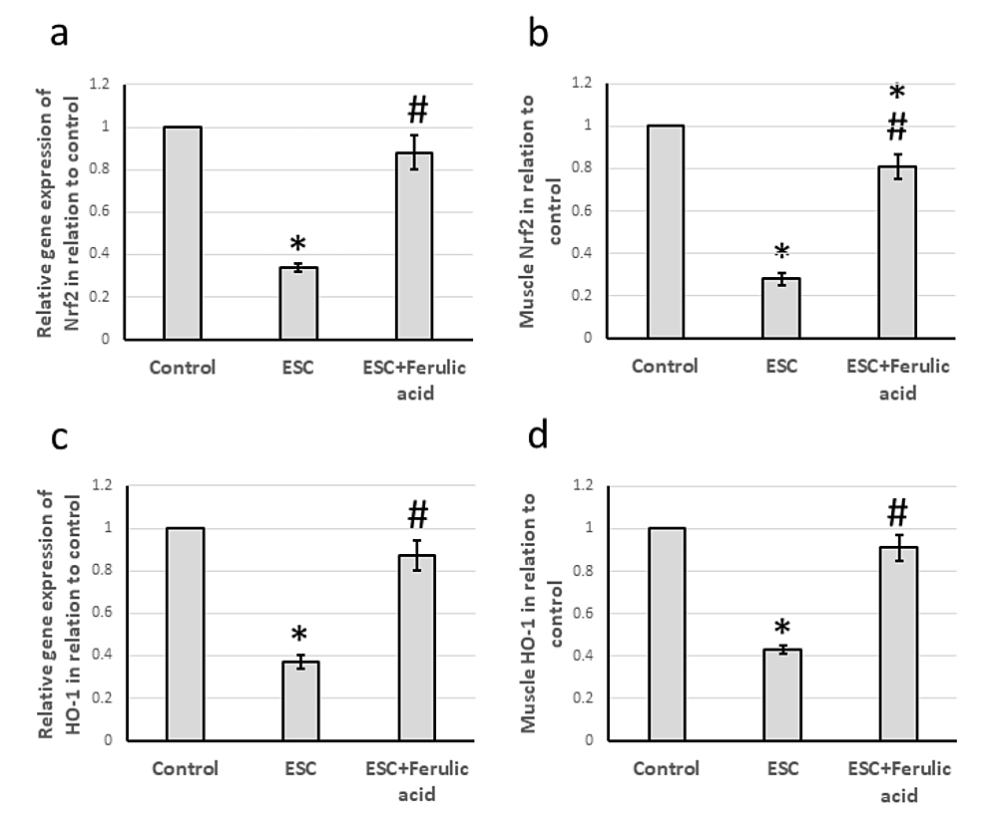
Effect of ESC and 50 mg/kg ferulic acid on gene expression of Nrf2 (a) and HO-1 (c) as well as the protein level of Nrf2 (b) and HO-1 (d). * significant difference against the control group at p<0.05. # significant difference against the ESC group at p<0.05. ESC, Ehrlich solid carcinoma, Nrf2; nuclear factor erythroid 2–related factor 2; HO-1, heme oxygenase-1.

Our study revealed significant decreases in expression levels of Nrf2 and HO-1 by 66% and 63%, respectively, in ESC rats compared to the control group. Additionally, we observed a decline of 72% and 57% in the muscle levels of Nrf2 and HO-1, respectively, in the ESC rats as compared to the control group. However, we found that administering ferulic acid to the ESC rats led to a significant increase in gene expression and muscle protein levels of both Nrf2 and HO-1, though the levels remained lower than those in the control group (Figure 4).

Effect of ferulic acid on ESC-induced elevation in the expression of cMyc and cyclin D1

**Figure 5:**
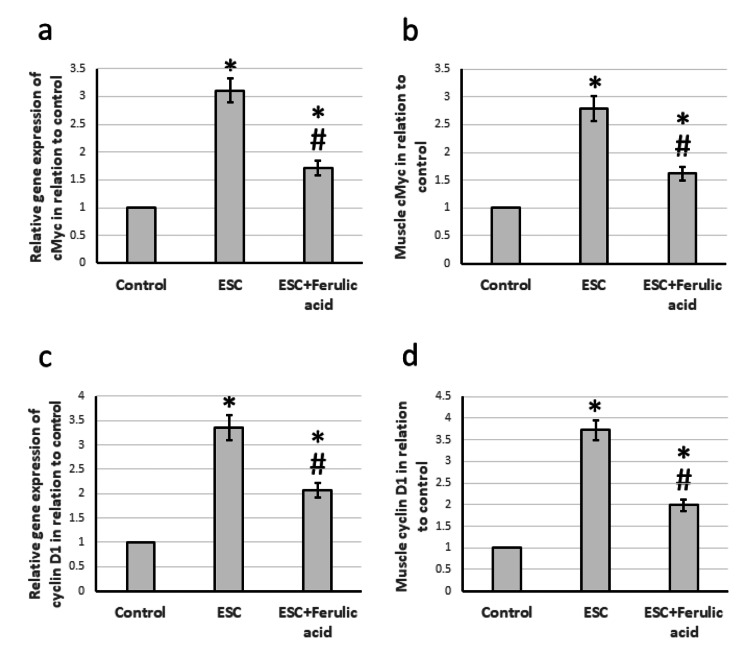
Effect of ESC and 50 mg/kg ferulic acid on gene expression of cMyc (a) and cyclin D1 (c) as well as the protein level of cMyc (b) and cyclin D1 (d). * significant difference against the control group at p<0.05. # significant difference against the ESC group at p<0.05. ESC, Ehrlich solid carcinoma.

The muscle samples taken from rats with ESC displayed a significant increase in gene expression of cMyc and cyclin D1, at 3.11 and 3.36 times higher than the control group. Examination of muscle levels also showed a notable increase in protein levels of both cMyc and cyclin D1, at 2.79 and 3.36 times higher than the control rats. However, treatment of ESC rats with ferulic acid helped reduce the over-expression of cMyc and cyclin D1, although levels still remained higher than in the control group (Figure 5).

Effect of ferulic acid on ESC-induced elevation in the expression of mTOR and STAT3

**Figure 6:**
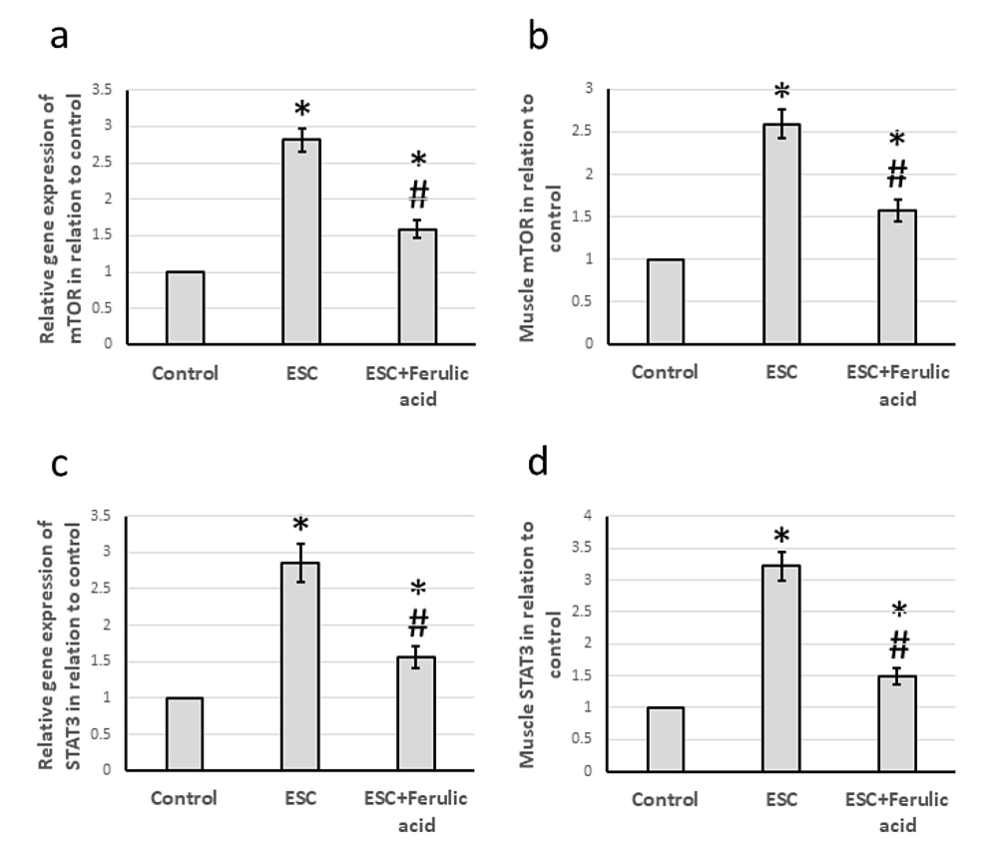
Effect of ESC and 50 mg/kg ferulic acid on gene expression of mTOR (a) and STAT3 (c) as well as the protein level of mTOR (b) and STAT3 (d). * Significant difference against the control group at p<0.05. # significant difference against the ESC group at p<0.05. ESC, Ehrlich solid carcinoma; mTOR, mammalian target of rapamycin; STAT3, signal transducer and activator of transcription 3.

The results showed that there was a significant increase in the expression of the mTOR and STAT3 genes by 2.81-fold and 2.86-fold, respectively, in rats treated with ESC compared to control rats. Additionally, there was a 2.59-fold and 2.86-fold increase in the protein levels of mTOR and STAT3 in the muscles of ESC rats compared to control rats. However, treatment of ESC rats with ferulic acid helped to reduce the overexpression of both genes (Figure 6).

## Discussion

As per the 2018 report by the World Health Organization, cancer is the second leading cause of death worldwide, responsible for over 9.6 million fatalities. Unfortunately, this number is predicted to rise to 21.4 million by 2030. Cancer treatment often causes severe symptoms that can lead to emotional distress, physical limitations, and depression, all of which negatively impact a patient's quality of life [18]. Furthermore, cancer chemotherapy is expensive and can result in serious side effects. Therefore, cancer is considered the primary cause of mortality and a reduction in life expectancy globally. To tackle this issue, we researched the potential chemotherapeutic effects of ferulic acid. For our research, we utilized ESC, a rapidly growing model of cancer. When implanted intraperitoneally or subcutaneously, Ehrlich cancer cells can develop into ascites and solid forms. In our study, we implanted Ehrlich cells subcutaneously in the thigh of the left hind leg, resulting in the appearance of a lump that gradually grew over several weeks due to the progression of the tumor. To confirm the diagnosis, we examined micro-images of the separated tumor stained with toluidine blue. The images showed clear evidence of malignant cells with a high nuclear-cytoplasmic ratio, clumped, roped, and stripling chromatin, indicating diffuse infiltrative positive staining.

In our search for a chemotherapeutic agent, we investigated the effectiveness of ferulic acid, which has been previously documented to have antitumor activities through various mechanisms [6-8]. Our findings indicate that ferulic acid significantly reduces tumor volume and weight compared to the control group. Additionally, it increases the mean survival time of ESC rats from 27 days to 49 days. Microscopic examination of ESC rats treated with ferulic acid also revealed an improvement in cell structure. Our study aimed to uncover a new mechanism for the antitumor activity of ferulic acid by targeting HIF-1α and observing its effects on antioxidant proteins, proliferation markers, and cellular regulators.

HIF-1α is a protein with a short half-life that serves as a primary marker of hypoxia. When activated, it can induce cancer tumorigenicity and progression. The protein has two subunits, HIF-1α and HIF-1β, with HIF-1α being strictly controlled by hypoxia and only activated during hypoxic conditions. During hypoxia, the HIF-1α protein is activated and moves into the nucleus, where it is linked to the DNA to help the cell adjust its metabolism process to the new situation [19]. Normal cells have a small and undetectable amount of HIF-1α, while it is highly activated in many tumors. Our research found a significant increase in HIF-1α expression in ESC, which was downregulated after ferulic acid treatment. However, no previous study has illustrated the effect of ferulic acid on HIF-1α in ESC.

Our focus was on the impact of ESC and ferulic acid treatment on the protective antioxidant systems within the cell to counteract the harmful effects of oxidative stress caused by HIF-1α. Specifically, we looked at Nrf2 and HO-1, which play crucial roles in preventing cancer development and progression by regulating cell proliferation, apoptosis, and cell cycle. When the body is under oxidative stress, it activates stress response molecules like Nrf2 and HO-1 to combat the harmful effects of reactive oxygen species generated in all body cells [20]. Nrf2 is a tumor suppressor gene that protects cells from oxidative and electrophilic attacks, making it anti-carcinogenic. Studies have shown that Nrf2 has a protective function in various diseases, including cancer, neurological diseases, cardiovascular problems, aging, inflammation, and photo-oxidative stress. HO-1 is one of the antioxidant defense proteins downstream of Nrf2 and is cyto-protective [21]. Our research found that ferulic acid could reverse the ESC-induced reduction in the expression of Nrf2 and HO-1 that was associated with improvement in the muscle tissue structure, as indicated in the stained micro-images. Ferulic acid enhances the translocation of Nrf2 into the nucleus to promote its antioxidant protective effects [22]. Many experimental disease models have shown that ferulic acid activates Nrf2 and HO-1, but no previous research has illustrated its effect on any cancer type.

Our research focused on exploring how ferulic acid affects the proliferation markers cMyc and cyclin D1, which are key players in tumorigenesis. As cell proliferation is a crucial element in the development of tumors, we aimed to investigate the impact of ferulic acid on these markers. The c-Myc transcription factor is a well-known oncogene that can accelerate cell cycle progression and is upregulated in various malignant tumors [23]. Similarly, cyclin D1 is a proto-oncogene that is overexpressed in different types of cancer and can cause uncontrolled cell proliferation [24]. Our study found that ferulic acid significantly reduced the activation of both cMyc and cyclin D1, leading to antitumor activity and a decrease in tumor weight and size. Previous studies have also reported that ferulic acid inhibits the expression of cyclin D1, reducing cell growth, and rescuing the expression of cyclin D1 in various cancer types [7,25]. However, our study is the first to demonstrate ferulic acid's ability to reduce the expression of cMyc and cyclin D1 in ESC.

Our study focused on analyzing the effects of ferulic acid on cell function by investigating the roles of mTOR and STAT3. mTOR, a key factor in autophagy induction, plays a crucial role in cell growth, proliferation, autophagy, and survival. Promising results have been observed in mTOR inhibitor tests against tumor cells. STAT3 is a transcriptional factor that mTOR targets directly, leading to STAT3 phosphorylation. Additionally, the mTOR inhibitor rapamycin can suppress STAT3 phosphorylation [26]. STAT3 is involved in immune responses, stem cell maintenance, and tumorigenesis. It transfers signals from outside the cell to the nucleus, activating the transcription of downstream oncogenes like Myc [27]. Abnormal STAT3 activation is a critical factor in the occurrence and development of cancer by promoting malignant cell proliferation, migration, and metastasis. Studies have shown that high STAT3 activity or phosphorylated STAT3 activation is associated with poor overall survival and unfavorable progression-free survival in ovarian cancer patients. Moreover, persistent STAT3 activation can promote tumor progression and metastasis in various cancers [28]. Our research indicates that treating with ferulic acid significantly reduces the expression of mTOR and STAT3, leading to a decrease in the tumor. Previous studies have shown that ferulic acid inhibits mTOR, resulting in the inhibition of proliferation and metastasis in human lung cancer cells [29]. However, our study is the first to demonstrate that ferulic acid can reduce the expression of mTOR and STAT3 in ESC.

**Figure 7:**
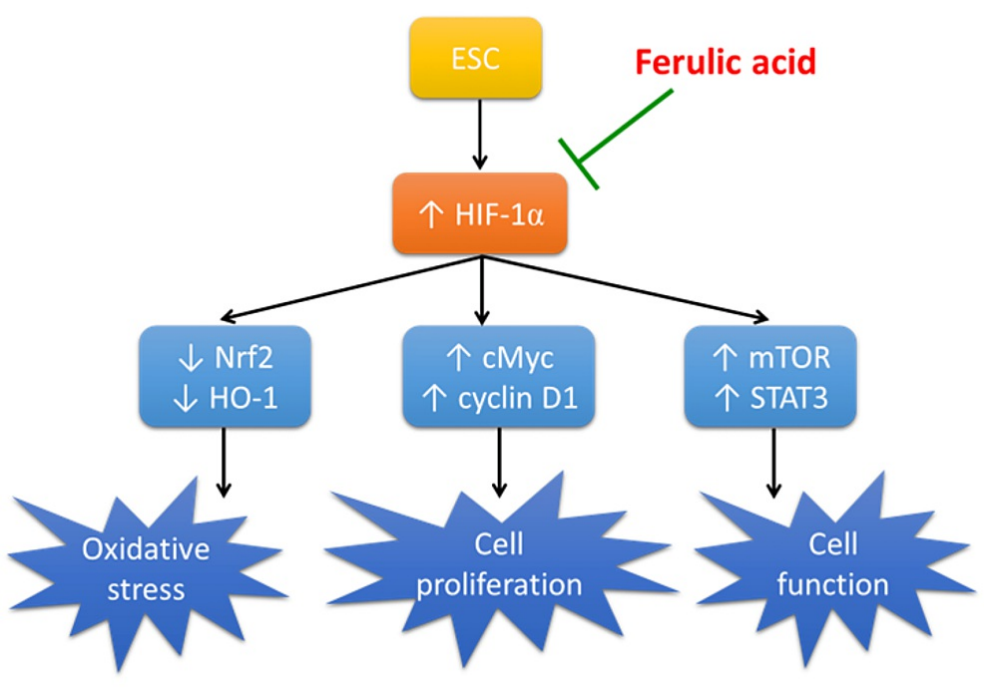
Schematic presentation of the mechanism of action of ferulic acid in ESC. ESC, Ehrlich solid carcinoma; cMyc, cellular Myc; HO-1, heme oxygenase-1; HIF-1α, hypoxia-inducible factor; mTOR, mammalian target of rapamycin; Nrf2, nuclear factor erythroid 2–related factor 2; STAT3, signal transducer and activator of transcription 3. Image Credits: The authors of the manuscript.

Ferulic acid is an encouraging chemotherapy option owing to its natural origin, cost-effectiveness, and safety. The mechanism of its therapeutic impact in ESC is outlined in Figure 7. Nonetheless, current research has certain constraints. For example, rats have distinct metabolic processes than humans, which may result in diverse drug effects. Furthermore, there are various animal models for cancer induction, but our study only used one approach.

## Conclusions

Our study aimed to explore whether ferulic acid has potential chemotherapeutic properties against ESC. Our findings indicate that ferulic acid can reduce tumor size and weight while improving the structure of muscle cells, suggesting it may have antineoplastic activity against ESC. Further investigation revealed that ferulic acid downregulates HIF-1α, leading to increased expression of antioxidant proteins Nrf2 and HO-1. Additionally, ferulic acid decreases the expression of proliferation markers cMyc and cyclin D1 and downregulates cellular regulators mTOR and STAT3.
